# Supplementary material for: Retrodiction beyond the Heisenberg uncertainty relation
Source: Nat Commun. 2020 Nov 9;11:5658. doi: 10.1038/s41467-020-19495-1 (PMC7652952; doi:10.1038/s41467-020-19495-1)
Supplement: Supplementary file 1 — Supplementary Information [file 41467_2020_19495_MOESM1_ESM.pdf]

Supplementary Information for
Retrodiction beyond the Heisenberg uncertainty relation

Bao and Jin *et al.*

Supplementary Note 1. Light-atom interaction

In the limit of large laser detuning from the optical transition, one can disregard absorption in the interaction of light with the atomic $D2$ transition from the $5S_{1/2}, F=2$ ground state to the $5P_{3/2}$ excited state and employ the effective interaction Hamiltonian [1]

$$\begin{aligned}\hat{H}_{\text{int}} = -\frac{\hbar c\Gamma}{8A\Delta}\frac{\lambda^2}{2\pi}\int_0^L &\{a_0\hat{\Phi}(z,t) + a_1\hat{S}_{\text{z}}(z,t)\hat{j}_{\text{z}}(z,t) \\ &+ a_2[\hat{\Phi}(z,t)\hat{j}_{\text{z}}^2(z,t) - \hat{S}_-(z,t)\hat{j}_+^2(z,t) \\ &- \hat{S}_+(z,t)\hat{j}_-^2(z,t)]\}n_{\text{A}}A dz,\end{aligned} \tag{1}$$

where $\lambda = 780$ nm and $\Gamma = 2\pi\times 6.067$ MHz are the wavelength of the probe light and the full width at half maximum (FWHM) linewidth of the excited state, respectively, while n_{A} is the atomic density. A is the cross section of the laser beam and Φ is the photon flux. The first scalar term can be treated as a DC Stark shift, which equally shifts all atomic energy levels and is proportional to the photon flux or light intensity. The second vector term provides the desired Faraday rotation operation where the atomic spin $\mathbf{J}$ is rotated around the z-axis by an amount proportional to $\hat{S}_{\text{z}}$. Likewise, the Stokes vector $\mathbf{S}$ is rotated around the z-axis by an amount proportional to $\hat{J}_{\text{z}}$. The last tensor term gives rise to a complicated dynamical Stark shift which is useful in some quantum protocols and will vanish for large detunings with respect to the hyperfine splitting of the excited state, as the coefficient a_2 goes to zero. For the D2 transition starting from the hyperfine ground state $F=2$, the detuning dependent vector and tensor polarizabilities a_1, and a_2 are given by [1, 2]

$$a_1 = \frac{\sqrt{2}}{100}\left(-\frac{15}{1-\Delta_{13}/\Delta} - \frac{25}{1-\Delta_{23}/\Delta} + 140\right), \tag{2}$$

$$a_2 = \frac{\sqrt{2}}{40}\left(\frac{1}{1-\Delta_{13}/\Delta} - \frac{5}{1-\Delta_{23}/\Delta} + 4\right), \tag{3}$$

where $\Delta_{13} = 2\pi\times 423.60$ MHz and $\Delta_{23} = 2\pi\times 266.65$ MHz are the hyperfine splitting in the ^{87}Rb excited state $5P_{3/2}$, respectively, while Δ is the laser detuning with respect to $5P_{3/2}F' = 3$.

Given $\Delta = -2\pi\times 2.09$ GHz (negative sign represents the blue detuning) in our experiment, $\frac{a_2}{a_1} = 0.0094 \ll 1$, thus, the tensor interaction can be ignored in our experiment, and the dispersive interaction of light with the atomic D2 transition can be approximated by the quantum non-demolition (QND)-type Faraday interaction Hamiltonian,

$$\hat{H}_{\text{int}} = \alpha\hat{J}_{\text{z}}\hat{S}_{\text{z}}, \tag{4}$$

with $\alpha = -\frac{\Gamma}{8A\Delta}\frac{\lambda^2}{2\pi}a_1$.

To describe the interaction between light and atoms in the experiment, the quantum state of light is characterized by the Stokes operators $\hat{S}_{\text{x}}$, $\hat{S}_{\text{y}}$ and $\hat{S}_{\text{z}}$, which are given by the differences of the number operators of the photons polarized in different orthogonal bases. For light propagating in the z-direction, we have

$$\hat{S}_{\text{x}} = \frac{1}{2}(\hat{a}_{\text{x}}^\dagger\hat{a}_{\text{x}} - \hat{a}_{\text{y}}^\dagger\hat{a}_{\text{y}}), \tag{5}$$

$$\hat{S}_{\text{y}} = \frac{1}{2}(\hat{a}_{+45^\circ}^\dagger\hat{a}_{+45^\circ} - \hat{a}_{-45^\circ}^\dagger\hat{a}_{-45^\circ}) = \frac{1}{2}(\hat{a}_{\text{x}}^\dagger\hat{a}_{\text{y}} + \hat{a}_{\text{y}}^\dagger\hat{a}_{\text{x}}), \tag{6}$$

$$\hat{S}_{\text{z}} = \frac{1}{2}(\hat{a}_{\sigma^+}^\dagger\hat{a}_{\sigma^+} - \hat{a}_{\sigma^-}^\dagger\hat{a}_{\sigma^-}) = \frac{1}{2i}(\hat{a}_{\text{x}}^\dagger\hat{a}_{\text{y}} - \hat{a}_{\text{y}}^\dagger\hat{a}_{\text{x}}), \tag{7}$$

with the creation and annihilation operators $\hat{a}^\dagger_{\text{x,y},\pm45^\circ,\sigma^\pm}$, $\hat{a}_{\text{x,y},\pm45^\circ,\sigma^\pm}$ in x, y, $\pm45^\circ$ and $\sigma^\pm$- polarization. The Stokes vector satisfies the angular momentum commutator relation, $[\hat{S}_{\text{y}}, \hat{S}_{\text{z}}] = i\hat{S}_{\text{x}}$ which implies the Heisenberg uncertainty Heisenberg uncertainty relation $\text{Var}(\hat{S}_{\text{y}})\cdot\text{Var}(\hat{S}_{\text{z}}) \geq \frac{\langle S_{\text{x}}\rangle^2}{4}$. In the y- linearly polarized coherent light pulses to interact with the atomic ensembles, which means S_{x} can be treated as a large classical value proportional to the photon flux

$\Phi = P/(\hbar\omega)$ where P represents the optical power and $\hbar\omega$ is the energy of a single photon. and $\hbar\omega$ is the energy of a single photon. The quantum variables $\hat{S}_{\rm y}$ and $\hat{S}_{\rm z}$ are the physical variables we are The coupling constant in the main text is defined as $\kappa^2 = \frac{1}{2}\alpha^2 J_{\rm x}\Phi\tau$. Here τ is the probe pulse duration.

Supplementary Note 2. Stroboscopic back-action-evading measurement

Given a large enough laser detuning, the relation $\left[\hat{H}_{\rm int}, \hat{J}_{\rm z}\right] = 0$ ensures the QND nature of the measurement with respect to the interaction Hamiltonian. Under the influence of a magnetic field along the x-axis, however, the Zeeman Hamiltonian $\hbar\Omega_{\rm L}\hat{J}_{\rm x}$ couples $\hat{J}_{\rm z}$ and $\hat{J}_{\rm y}$. To restore the QND property of the probing scheme in the presence of the Zeeman induced precession of the spin at the Larmor frequency $\Omega_{\rm L}$, we probe the ensemble with δ-pulses at the times $t = 0, \frac{\pi}{\Omega_{\rm L}}, ..., \frac{n\pi}{\Omega_{\rm L}}$, where the precession does not mix $\hat{J}_{\rm y}$ and $\hat{J}_{\rm z}$. In such an ideal setup, only the shot noise in the light affects the precision of the measurement of $\hat{J}_{\rm z}$, while the conjugate quadrature $\hat{J}_{\rm y}$ becomes anti-squeezed due to the measurement backaction.

We introduce canonical operators for light

$$\hat{x}_{\rm L} = \frac{\hat{S}_{\rm y}}{\sqrt{|\langle S_{\rm x}\rangle|}}, \hat{p}_{\rm L} = \frac{\hat{S}_{\rm z}}{\sqrt{|\langle S_{\rm x}\rangle|}}, \tag{8}$$

and for the collective spin of atoms

$$\hat{x}_{\rm A} = \frac{\hat{J}_{\rm y}}{\sqrt{|\langle J_{\rm x}\rangle|}}, \hat{p}_{\rm A} = \frac{\hat{J}_{\rm z}}{\sqrt{|\langle J_{\rm x}\rangle|}}, \tag{9}$$

obeying $[\hat{x}_{\rm L}, \hat{p}_{\rm L}] = i$ and $[\hat{x}_{\rm A}, \hat{p}_{\rm A}] = i$ ($\hbar = 1$) [1].

The input-output relations of the matter-light interaction is then given by

$$\begin{pmatrix} \hat{x}_{\rm L}^{\rm out} \\ \hat{p}_{\rm L}^{\rm out} \\ \hat{x}_{\rm A}^{\rm out} \\ \hat{p}_{\rm A}^{\rm out} \end{pmatrix} = \begin{pmatrix} 1 & 0 & 0 & \kappa \\ 0 & 1 & 0 & 0 \\ 0 & \kappa & 1 & 0 \\ 0 & 0 & 0 & 1 \end{pmatrix} \begin{pmatrix} \hat{x}_{\rm L}^{\rm in} \\ \hat{p}_{\rm L}^{\rm in} \\ \hat{x}_{\rm A}^{\rm in} \\ \hat{p}_{\rm A}^{\rm in} \end{pmatrix}. \tag{10}$$

According to the formula for the variance of a Gaussian distributed variable $\hat{p}_{\rm A}$ conditioned on the measurement of another correlated Gaussian variable $\hat{x}_{\rm L}$, we have

$$\mathrm{Var}\left[\hat{p}_{\rm A}\right]\Bigg|_{\hat{x}_{\rm L}} = \mathrm{Var}\left[\hat{p}_{\rm A}\right] - \frac{\mathrm{Cov}^2\left[\hat{p}_{\rm A}, \hat{x}_{\rm L}\right]}{\mathrm{Var}\left[\hat{x}_{\rm L}\right]}, \tag{11}$$

where $\mathrm{Cov}\left[\hat{p}_{\rm A}, \hat{x}_{\rm L}\right]$ represents the covariance of $\hat{x}_{\rm L}$ and $\hat{p}_{\rm A}$, and following Supplementary Equation (10) we can calculate the conditional variance of $\hat{p}_{\rm A}$ as

$$\mathrm{Var}\left[\hat{p}_{\rm A}\right]\Bigg|_{\hat{x}_{\rm L}} = \frac{1}{1+\kappa^2}\frac{1}{2}, \tag{12}$$

where we assume coherent initial states for the light and atoms.

While this results fully accounts for the conditional spin squeezing by the measurement back action, we shall provide a theoretical description of the same result by means of the POVM formalism in the next section. That formalism is needed to account for the correlations between subsequent measurements on the same system.

We note that the QND character of the stroboscopic probing scheme only applies for infinitely short pulses (duty factor $D = 0$). In our experiments, we use finite duration probe pulses which implies a (small) spin rotation during the probing. This reduces the effective coupling to the desired spin component and couples the squeezed and anti-squeezed components and thus reduces the effect of squeezing. We shall return to an effective analysis of these effects at the end of Supplementary Note 3.

Supplementary Note 3. Past quantum Gaussian state in Faraday interaction

In terms of the canonical variables, the QND interaction that measures $\hat{p}_{\rm A}$ is similar to a continuous variable control-Z gate [3] and can be written as

$$\hat{C}_{\rm z} = e^{-i\kappa\hat{p}_{\rm A}\otimes\hat{p}_{\rm L}}. \tag{13}$$

The initial state of atom and light, Ψ_{A}^{in} and Ψ_{L}^{in}, can be expanded in the eigenstates of $\hat{p}_{\mathrm{A}}$ and $\hat{x}_{\mathrm{L}}$

$$\hat{p}_{\mathrm{A}}|a\rangle_{\hat{p}_{\mathrm{A}}} = a|a\rangle_{\hat{p}_{\mathrm{A}}}, \tag{14}$$

$$\hat{x}_{\mathrm{L}}|l\rangle_{\hat{x}_{\mathrm{L}}} = l|l\rangle_{\hat{x}_{\mathrm{L}}}, \tag{15}$$

with the wave functions

$$\Psi_{\mathrm{A}}^{in} = \int \psi_{\hat{p}_{\mathrm{A}}}(a)|a\rangle_{\hat{p}_{\mathrm{A}}} da, \tag{16}$$

$$\Psi_{\mathrm{L}}^{in} = \int \psi_{\hat{x}_{\mathrm{L}}}(l)|l\rangle_{\hat{x}_{\mathrm{L}}} dl. \tag{17}$$

The coherent states of collective spin and light are described by Gaussian wave functions $\psi_{\hat{p}_{\mathrm{A}}}(a)$ and $\psi_{\hat{x}_{\mathrm{L}}}(l)$ with a variance of $\frac{1}{2}$,

$$\psi_{\hat{p}_{\mathrm{A}}}(a) = \frac{1}{\pi^{1/4}} \exp(-\frac{a^2}{2}), \tag{18}$$

$$\psi_{\hat{x}_{\mathrm{L}}}(l) = \frac{1}{\pi^{1/4}} \exp(-\frac{l^2}{2}). \tag{19}$$

Applying the QND interaction to the initial state of atom and light, we get

$$\begin{aligned} \hat{C}_{\mathrm{z}}(\Psi_{\mathrm{A}}^{in} \otimes \Psi_{\mathrm{L}}^{in}) &= \hat{C}_{\mathrm{z}}[(\int \psi_{\hat{p}_{\mathrm{A}}}(a)|a\rangle_{\hat{p}_{\mathrm{A}}} da) \otimes (\int \psi_{\hat{x}_{\mathrm{L}}}(l)|l\rangle_{\hat{x}_{\mathrm{L}}} dl)] \\ &= \int\int \psi_{\hat{p}_{\mathrm{A}}}(a)\psi_{\hat{x}_{\mathrm{L}}}(l)(|a\rangle_{\hat{p}_{\mathrm{A}}} \otimes |l+\kappa a\rangle_{\hat{x}_{\mathrm{L}}}) da dl. \end{aligned} \tag{20}$$

After the interaction we measure the quadrature of light in the $\hat{x}_{\mathrm{L}}$ basis with the outcome m. The resulting conditional state of the atomic variables is updated as

$$\begin{aligned} \Psi_{\mathrm{A}}^{out} &\propto \int \psi_{\hat{p}_{\mathrm{A}}}(a)\psi_{\hat{x}_{\mathrm{L}}}(m-\kappa a)|a\rangle_{\hat{p}_{\mathrm{A}}} da \\ &\propto \int \exp[-\frac{(a-\frac{\kappa m}{1+\kappa^2})^2}{2/(1+\kappa^2)}]|a\rangle_{\hat{p}_{\mathrm{A}}} da. \end{aligned} \tag{21}$$

The mean value is displaced from 0 to $\frac{\kappa m}{1+\kappa^2}$, and as we saw in Supplementary Equation (12), the atomic variance of $\hat{p}_{\mathrm{A}}$ is reduced by a factor of $1+\kappa^2$.

3.1. Measurement operators

In the positive-operator valued measure (POVM) formalism, the initial atomic state is represented by the density matrix ρ_0, which can describe both pure and mixed states. When the QND interaction acts on the whole system-meter state ("system" = atoms, "meter" = light), the total density matrix $\rho_0 \otimes |\Psi_{\mathrm{L}}^{in}\rangle\langle\Psi_{\mathrm{L}}^{in}|$ is transformed into $\rho_{\mathrm{tot}} = \hat{C}_{\mathrm{z}}\left(\rho_0 \otimes |\Psi_{\mathrm{L}}^{in}\rangle\langle\Psi_{\mathrm{L}}^{in}|\right)\hat{C}_{\mathrm{z}}^{\dagger}$. By inserting the identity operator of the light $\hat{I}_{\mathrm{m}} = \sum_{l\in M} |l\rangle\langle l|_{\hat{x}_{\mathrm{L}}}$ we obtain

$$\rho_{\mathrm{tot}} = \sum_{m,m'\in M} \hat{\Omega}_{\mathrm{m}}\rho_0\hat{\Omega}_{m'}^{\dagger} \otimes |m\rangle\langle m'|_{\hat{x}_{\mathrm{L}}}, \tag{22}$$

where we have introduced the system operators $\hat{\Omega}_{\rm m} = (\hat{I}_{\rm A} \otimes \langle m|_{\hat{x}_{\rm L}})\hat{C}_{\rm z}(\hat{I}_{\rm A} \otimes |\Psi_{\rm L}^{in}\rangle)$. To calculate the effect of $\hat{\Omega}_{\rm m}$ on the atomic system, we insert $\int |a\rangle\langle a|_{\hat{p}_{\rm A}} da$ for the second occurrence of $\hat{I}_{\rm A}$,

$$\begin{aligned}
\hat{\Omega}_{\rm m} &= \hat{I}_{\rm A} \otimes \langle m|_{\hat{x}_{\rm L}} e^{-i\kappa\hat{p}_{\rm A}\otimes\hat{p}_{\rm L}} \int |a\rangle\langle a|_{\hat{p}_{\rm A}} da \otimes |\Psi_{\rm L}^{in}\rangle \\
&= \hat{I}_{\rm A} \otimes \langle m|_{\hat{x}_{\rm L}} \int |a\rangle\langle a|_{\hat{p}_{\rm A}} e^{-i\kappa a\hat{p}_{\rm L}} |\Psi_{\rm L}^{in}\rangle da \\
&= \int |a\rangle\langle a|_{\hat{p}_{\rm A}} \langle m|_{\hat{x}_{\rm L}} e^{-i\kappa a\hat{p}_{\rm L}} |\Psi_{\rm L}^{in}\rangle da \\
&= \int \left[|a\rangle\langle a|_{\hat{p}_{\rm A}} \langle m|_{\hat{x}_{\rm L}} e^{-i\kappa a\hat{p}_{\rm L}} \int \psi_{\hat{x}_{\rm L}}(l)|l\rangle_{\hat{x}_{\rm L}} dl \right] da \\
&= \int \left[|a\rangle\langle a|_{\hat{p}_{\rm A}} da \langle m|_{\hat{x}_{\rm L}} \int \psi_{\hat{x}_{\rm L}}(l)|l+\kappa a\rangle_{\hat{x}_{\rm L}} dl \right] da \\
&= \int \psi_{\hat{x}_{\rm L}}(m-\kappa a)|a\rangle\langle a|_{\hat{p}_{\rm A}} da.
\end{aligned} \tag{23}$$

Similarly, the operator for measuring the rotated quadrature observable $\hat{x}_{\rm A}(\theta) = \hat{p}_{\rm A}\cos\theta + \hat{x}_{\rm A}\sin\theta$ with outcome m is

$$\hat{\Omega}_{\rm m} = \int \psi_{\hat{x}_{\rm L}}(m-\kappa a)|a,\theta\rangle\langle a,\theta|{\rm d}a, \tag{24}$$

where $|a,\theta\rangle$ is the eigenstate of $\hat{x}_{\rm A}(\theta)$ with eigenvalue a. In the limit of large κ, $\hat{\Omega}_{\rm m}$ becomes a (strong) projective measurement $\hat{\Omega}_{\rm a} = |a,\theta\rangle\langle a,\theta|$, with $a = m/\kappa$.

3.2. Retrodiction beyond the Heisenberg uncertainty relation

We consider two types of measurements: a hypothetical projective measurement $\hat{\Omega}_a$, with outcome a for the $\hat{x}_{\rm A}(\theta)$ and the actually performed optical measurement $\hat{\Omega}_{m_2}$, with outcome m_2 for the observable $\hat{x}_{\rm L}$.

The outcome of these measurement can be predicted by the density matrix conditioned on the first measurement outcome m_1, and they can be retrodicted by incorporating also the outcome of later optical measurements of $\hat{x}_{\rm A}$ and $\hat{p}_{\rm A}$ with outcomes m_3 and m_4.

Conditioned on the first measurement outcome m_1, i.e. by using the the prediction only, if the 2nd pulse is a hypothetical projective measurement $\hat{\Omega}_a$, we have

$$\begin{aligned}
\Pr(a|m_1) &= \frac{{\rm Tr}\left(\hat{\Omega}_a\rho\hat{\Omega}_a^\dagger\right)}{\int {\rm Tr}\left(\hat{\Omega}_{a'}\rho\hat{\Omega}_{a'}^\dagger\right) da'} \\
&\propto \langle a,\theta|\rho|a,\theta\rangle,
\end{aligned} \tag{25}$$

where $\rho = \hat{\Omega}_{m_1}\rho_0\hat{\Omega}_{m_1}^\dagger$ is the density matrix after 1st pulse measurement. If the 2nd measurement is a POVM $\hat{\Omega}_{m_2}$, we have

$$\begin{aligned}
\Pr(m_2|m_1) &= \frac{{\rm Tr}\left(\hat{\Omega}_{m_2}\rho\hat{\Omega}_{m_2}^\dagger\right)}{\int {\rm Tr}\left(\hat{\Omega}_{m_2'}\rho\hat{\Omega}_{m_2'}^\dagger\right) dm_2'} \\
&\propto \int \psi_{\hat{x}_{\rm L}}^2(m_2-\kappa a)\langle a,\theta|\rho|a,\theta\rangle dx.
\end{aligned} \tag{26}$$

The joint probability for all four outcomes $\{m_1, a, m_3, m_4\}$ with an atomic projective measurement or $\{m_1, m_2, m_3, m_4\}$ with only optical measurements can be described as

$$\Pr(m_1, M, m_3, m_4) = {\rm Tr}\left(\hat{\Omega}_{m_4}\hat{\Omega}_{m_3}\hat{\Omega}_m\hat{\Omega}_{m_1}\rho_0\hat{\Omega}_{m_1}^\dagger\hat{\Omega}_{\rm m}^\dagger\hat{\Omega}_{m_3}^\dagger\hat{\Omega}_{m_4}^\dagger\right). \tag{27}$$

For a projective measurement $\hat{\Omega}_{\rm m} = \hat{\Omega}_a$, the atomic state is projected to an atomic spin eigenstate $|a,\theta\rangle$, and the measurement result is the corresponding eigenvalue a. For an optical measurement $\hat{\Omega}_{\rm m} = \hat{\Omega}_{m_2}$, the measurement result is m_2 containing information about the light and atomic spin.

As a result of both the prediction and retrodiction, i.e. conditioned on the measurement outcomes m_1, m_3 and m_4, the probability for the outcome is thus given as

$$\begin{aligned}
\Pr(a|m_1,m_3,m_4) &= \frac{\mathrm{Tr}\left(\hat{\Omega}_a\rho\hat{\Omega}_a^\dagger E\right)}{\int \mathrm{Tr}\left(\hat{\Omega}_{a'}\rho\hat{\Omega}_{a'}^\dagger E\right)da'} \\
&\propto \langle a,\theta|\rho|a,\theta\rangle\langle a,\theta|E|a,\theta\rangle, && (28)\\
\Pr(m_2|m_1,m_3,m_4) &= \frac{\mathrm{Tr}\left(\hat{\Omega}_{m_2}\rho\hat{\Omega}_{m_2}^\dagger E\right)}{\int \mathrm{Tr}\left(\hat{\Omega}_{m_2'}\rho\hat{\Omega}_{m_2'}^\dagger E\right)dm_2'} \\
&\propto \int\int \psi_{\hat{x}_\mathrm{L}}(m_2-\kappa_2 a)\psi_{\hat{x}_\mathrm{L}}(m_2-\kappa_2 a')\langle a,\theta|\rho|a',\theta\rangle\langle a',\theta|E|a,\theta\rangle da da', && (29)
\end{aligned}$$

where the effect matrix E conditioned on the 3rd and 4th measurement equals

$$E = \Omega_{m_3}^\dagger\Omega_{m_4}^\dagger\Omega_{m_4}\Omega_{m_3}. \tag{30}$$

Using the expression for the POVM operators, we obtain

$$\begin{aligned}
E &= \left[\int \psi_{\hat{x}_\mathrm{L}}(m_3-\kappa_3 a_3')|a_3',0\rangle\langle a_3',0|\mathrm{d}a_3'\right]\cdot\left[\int \psi_{\hat{x}_\mathrm{L}}(m_4-\kappa_4 a_4)^2|a_4,\frac{\pi}{2}\rangle\langle a_4,\frac{\pi}{2}|\mathrm{d}a_4\right] \\
&\cdot\left[\int \psi_{\hat{x}_\mathrm{L}}(m_3-\kappa_3 a_3)|a_3,0\rangle\langle a_3,0|\mathrm{d}a_3\right] && (31)\\
&\propto \int \psi_{\hat{x}_\mathrm{L}}(m_3-\kappa_3 a_3')\left[\int \psi_{\hat{x}_\mathrm{L}}(m_3-\kappa_3 a_3)\left(\int \psi_{\hat{x}_\mathrm{L}}(m_4-\kappa_4 a_4)^2 e^{-ia_4(a_3-a_3')}\mathrm{d}a_4\right)|a_3',0\rangle\langle a_3,0|\mathrm{d}a_3\right]\mathrm{d}a_3'.
\end{aligned}$$

In order to obtain the elements $\langle a,\theta|\rho|a',\theta\rangle$ and $\langle a',\theta|E|a,\theta\rangle$ of ρ and E, we introduce the corresponding Wigner functions, cf. the definition,

$$W_{\rho(\mathrm{E})}(q,p) = \frac{1}{\pi}\int \langle p+r,0|\rho(\mathrm{E})|p-r,0\rangle e^{-2iqr}\mathrm{d}r. \tag{32}$$

The Wigner functions of ρ and E are

$$\begin{aligned}
W_\rho(q,p) &= \frac{1}{\pi}\int \langle p+r,0|\rho|p-r,0\rangle e^{-2iqr}\mathrm{d}r \\
&= \frac{1}{\pi}\exp\left(-\frac{(p-\frac{\kappa_1 m_1}{1+\kappa^2})^2}{1/(1+\kappa_1^2)} - \frac{q^2}{1+\kappa_1^2}\right), && (33)\\
W_\mathrm{E}(q,p) &= \frac{1}{\pi}\int \langle q+r,\frac{\pi}{2}|E|q-r,\frac{\pi}{2}\rangle e^{2ipr}\mathrm{d}r \\
&= \frac{\kappa_3\kappa_4}{\pi\sqrt{1+\kappa_3^2\kappa_4^2}}\exp\left(-\frac{(q-\frac{m_3}{\kappa_3})^2}{\frac{1}{\kappa_3^2}} - \frac{(p+\frac{m_4}{\kappa_4})^2}{\kappa_3^2+\frac{1}{\kappa_4^2}}\right), && (34)
\end{aligned}$$

while, e.g., $\langle p+r,0|\rho|p-r,0\rangle$ is the inverse Fourier transformation of $W_\rho(q,p)$,

$$\langle p+r,0|\rho|p-r,0\rangle = \frac{1}{\pi}\int W_\rho(q,p)e^{2iqr}\mathrm{d}q. \tag{35}$$

Introducing the rotated quadratures, $u = q\mathrm{cos}\theta - p\mathrm{sin}\theta$, $v = q\mathrm{sin}\theta + p\mathrm{cos}\theta$, we have the relations

$$\langle v,\theta|\rho|v',\theta\rangle = \frac{1}{\pi}\int W_\rho(q,p)e^{2iu(v-v')}\mathrm{d}u, \tag{36}$$

$$\langle v',\theta|E|v,\theta\rangle = \frac{1}{\pi}\int W_\mathrm{E}(q,p)e^{2iu(v'-v)}\mathrm{d}u, \tag{37}$$

where and p,q are eigenvalues of $\hat{p}_\mathrm{A}, \hat{x}_\mathrm{A}$ respectively.

Specifically, the diagonal elements are

$$\begin{aligned}\langle v,\theta|\rho|v,\theta\rangle &= \int W_\rho(q,p)\mathrm{d}u \\ &= \frac{1}{\sqrt{\pi}}\frac{1}{\sqrt{(1+\kappa_1^2)\mathrm{sin}^2\theta+\frac{\cos^2\theta}{1+\kappa_1^2}}}\exp\left(-\frac{v-\frac{k_1 m_1 \cos\theta}{1+\kappa_1^2}}{(1+\kappa_1^2)\mathrm{sin}^2\theta+\frac{\cos^2\theta}{1+\kappa_1^2}}\right), \end{aligned} \tag{38}$$

$$\begin{aligned}\langle v,\theta|E|v,\theta\rangle &= \int W_{\mathrm{E}}(q,p)\mathrm{d}u \\ &= \frac{1}{\sqrt{\pi}\sqrt{(\kappa_3^2+\frac{1}{\kappa_4^2})\cos^2\theta+\frac{1}{\kappa_3^2}\sin^2\theta}}\exp\left(-\frac{(v-\frac{m_3}{\kappa_3}\sin\theta+\frac{m_4}{\kappa_4}\cos\theta)^2}{(\kappa_3^2+\frac{1}{\kappa_4^2})\cos^2\theta+\frac{1}{\kappa_3^2}\sin^2\theta}\right). \end{aligned} \tag{39}$$

$\Pr(a|m_1)$, $\Pr(m_2|m_1)$, $\Pr(a|m_1,m_3,m_4)$ and $\Pr(m_2|m_1,m_3,m_4)$ are all Gaussian distributions determined by their respective mean values and variances.

By defining the variance of $\langle a,\theta|\rho|a,\theta\rangle$ and $\langle a,\theta|E|a,\theta\rangle$ as

$$\sigma_\rho^2 = f_\rho(\theta) = \frac{1}{2}\left(\frac{\cos^2\theta}{\kappa_1^2+1}+\left(\kappa_1^2+1\right)\sin^2\theta\right), \tag{40}$$

$$\sigma_{\mathrm{E}}^2 = f_{\mathrm{E}}(\theta) = \frac{1}{2}\left(\frac{\sin^2\theta}{\kappa_3^2}+\cos^2\theta\left(\kappa_3^2+\frac{1}{\kappa_4^2}\right)\right), \tag{41}$$

the associated variance of $\Pr(a|m_1)$ is

$$\begin{aligned}\mathrm{Var}(a|m_1) &= \sigma_\rho^2 \\ &= \frac{1}{2}\left(\frac{\cos^2\theta}{\kappa_1^2+1}+\left(\kappa_1^2+1\right)\sin^2\theta\right).\end{aligned} \tag{42}$$

The variance of $\Pr(m_2|m_1)$ is found to obey the relation

$$\begin{aligned}\mathrm{Var}(m_2|m_1) &= \frac{1}{2}+\kappa_2^2\sigma_\rho^2 \\ &= \frac{1}{2}+\kappa_2^2\mathrm{Var}(a|m_1),\end{aligned} \tag{43}$$

where the first term $\frac{1}{2}$ comes from optical shot noise.

The variance of $\Pr(a|m_1,m_3,m_4)$ is

$$\begin{aligned}\mathrm{Var}(a|m_1,m_3,m_4) &= \sigma_{\rho\mathrm{E}}^2 \\ &= (1/\sigma_\rho^2+1/\sigma_{\mathrm{E}}^2)^{-1} \\ &= \frac{1}{2}\cdot\frac{\left(\frac{\cos^2\theta}{\kappa_1^2+1}+\left(\kappa_1^2+1\right)\sin^2\theta\right)\left(\frac{\sin^2\theta}{\kappa_3^2}+\cos^2\theta\left(\kappa_3^2+\frac{1}{\kappa_4^2}\right)\right)}{\frac{\cos^2\theta}{\kappa_1^2+1}+(\kappa_1^2+1)\sin^2\theta+\frac{\sin^2\theta}{\kappa_3^2}+\cos^2\theta\left(\kappa_3^2+\frac{1}{\kappa_4^2}\right)}.\end{aligned} \tag{44}$$

We show the polar plot of $\sigma_{\rho\mathrm{E}}^2$ for different coupling strengths κ_1 and κ_3 in Supplementary Figure 1. Interestingly, while our initial argument was that prior and posterior measurements of two conjugate observables would in principle make a better estimation of both values than indicated by the Heisenberg uncertainty relation, no particular consequences were expected for quadratures along other directions. Rather than a squeezing ellipse, the retrodicted distribution shown in Supplementary Figure 1 suggests the form of a "squeezing butterfly" [4].

We can also obtain an analytical expressions for the variance of the optical measurement outcomes around the retrodicted expectation value, $\mathrm{Var}(m_2|m_1,m_3,m_4)$. This expression is more complex since it involves the off-diagonal elements of ρ and E in Supplementary Equation (28) and (29), and, in particular, it does not have a simple relationship with $\mathrm{Var}(a|m_1,m_3,m_4)$. This is reflected by the polar plots of $\frac{\mathrm{Var}(m_2|m_1,m_3,m_4)-\frac{1}{2}}{\kappa_2^2}$ in Supplementary Figure 2, which agrees with $\mathrm{Var}(a|m_1,m_3,m_4)$ for $\theta=0,\frac{\pi}{2}$ (the $\hat{p}_{\mathrm{A}}$ and $\hat{x}_{\mathrm{A}}$ quadratures), and for all quadratures in the large κ_2 (strong measurement) limit.

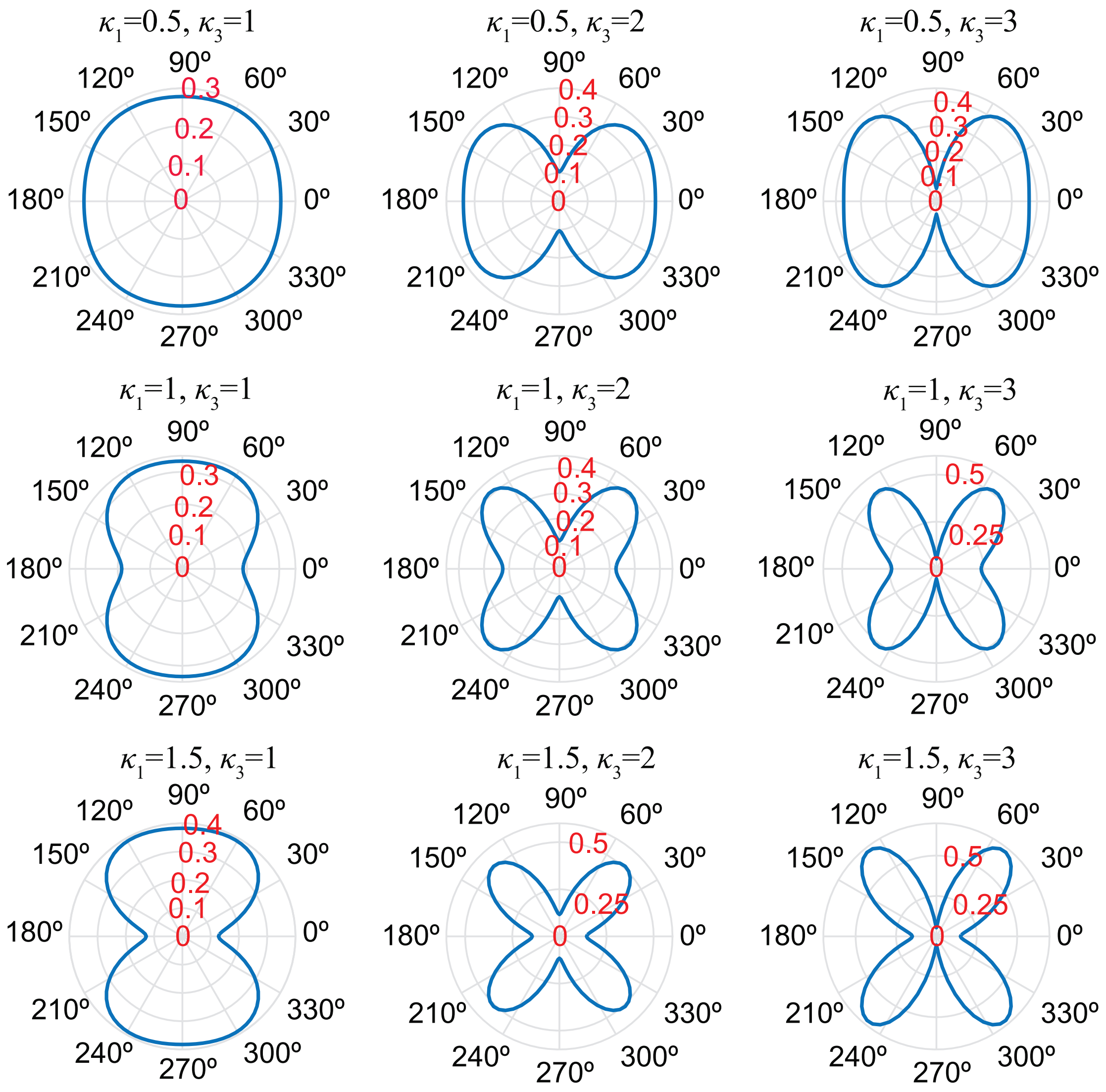

Supplementary Figure 1: Polar distribution of $\sigma^2_{\rho\mathrm{E}}$ for different coupling strengths κ_1 and κ_3 and fixed $\kappa_4^2 = 2$. 0.5 represents the standard quantum limit (SQL).

Supplementary Note 4. The need for the final measurement of $\hat{p}_{\mathrm{A}}$

If we probe only the $\hat{x}_{\mathrm{A}}$ component after the m_2 measurement, i.e. $\kappa_4 = 0$, $E = \Omega^\dagger_{m_3}\Omega_{m_3}$, $\langle a, \theta|E|a, \theta\rangle$ has infinite variance in all directions other than $\theta = \frac{\pi}{2}$. For all other directions, the post selection hence presents no reduction of the variance compared to the prediction by ρ when $\kappa_4 \to 0$. Notably, Supplementary Equation (44) reduces to

$$\sigma^2_{\rho\mathrm{E}(\kappa_4\to 0)} = \begin{cases} \sigma^2_\rho & \theta \neq \frac{\pi}{2} \\ \frac{1}{2}\cdot\frac{1+\kappa_1^2}{1+\kappa_3^2+\kappa_1^2\kappa_3^2} & \theta = \frac{\pi}{2} \end{cases} \tag{45}$$

This is shown with the distribution patterns of $\sigma^2_{\rho\mathrm{E}}$ around $\theta = \frac{\pi}{2}$ in Supplementary Figure 3 for different values of κ_4. To observe the variance reduction induced by retrodiction in a projective measurement, the atomic quadrature angle has to be controlled with high accuracy. For the retrodiction of the optical measurement, however, the probing of $\hat{p}_{\mathrm{A}}$ with strength κ_4 does not have a similarly important effect. This is shown in Supplementary Figure 4, for $\kappa_2^2 = 0.81$, where the variance shows a reduction in a broader range around $\theta = \frac{\pi}{2}$ even if $\kappa_4 = 0$. Supplementary Figure 4 shows that when we increase the value of κ_4 from 0, an improvement is observed. However, the improvement saturates around $\kappa_4 \simeq 1.5$. This agrees qualitatively with the experimental results.

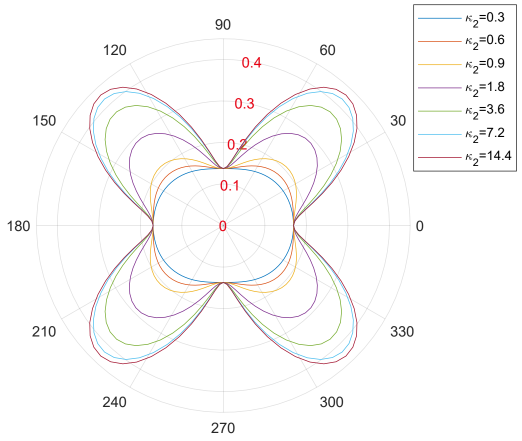

Supplementary Figure 2: Theoretical value of the (rescaled) variance of the optical measurement $\frac{\mathrm{Var}(m_2|m_1,m_3,m_4)-\frac{1}{2}}{\kappa_2^2}$, for different value of κ_2. For $\theta = 0, \frac{\pi}{2}$, the rescaling makes the result independent of κ_2. In the limit of $\kappa_2 \to \infty$, $\hat{\Omega}_{m_2}$ approaches the projective measurement $\hat{\Omega}_a$, and the rescaled optical variance equals the theoretical value for the atomic variance as shown by the fat dashed lines.

Supplementary Note 5. Effects of off-resonant atomic excitation and decay

So far in our analysis we have ignored effects of dissipation, in particular the spontaneous emission by the off-resonant excitation of the atomic excited states. These processes have three consequences: (i) they break the permutation symmetry of the ensemble which is equivalent to a reduction of the effective mean spin projection and hence a reduced collective coupling to the light, (ii) they break the correlation between the given atom and the rest of the ensemble, which is ultimately the cause of the squeezing of the collective spin, and (iii) the atoms decay at random into one of the spin states, contributing a random fluctuating term in the collective spin [5, 6].

These processes occur with a probability $\eta_\tau = h\frac{\tilde{\kappa}^2}{d}$, where h is a numerical factor of order unity that depends on the probe polarization and the light-atom detuning, and $d = n_\mathrm{A}\sigma_0 L$ is the optical depth on resonance given by the atomic density n_A, the absorption cross section on resonance σ_0, and the length of the cell L. For a finite duty factor in the stroboscopic detection, the coupling strength acquires an effective value $\tilde{\kappa}$ discussed in the next section.

During the first pulse there is hence a competition between the squeezing due to probing and the increasing spin fluctuations due to decay, explaining the existence of an optimal probing time τ_1. While decay during the later measurement during τ_3 does not alter the previous state, it causes a reduction of the strength κ_4 of the last measurements, which impacts the retrodiction according to Supplementary Equation (44) and may be the cause of the slow increase of the uncertainty product as function of τ_3 in Fig. 3(b) of the main text.

Supplementary Note 6. Effect of finite probe pulse duration

So far, we described the ideal stroboscopic detection with δ-function pulses of an effective QND spin observable, interrogated at twice the spin oscillator frequency. In the experiments, however, we apply finite duration pulses, described by a stroboscopic function $u(t)$ with a duty factor D, where

$$u(t) = \begin{cases} 1 \; : & -\frac{DT}{4} + kT \leq t \leq \frac{DT}{4} + kT \\ 0 \; : & \frac{DT}{4} + kT < t < -\frac{DT}{4} + (k+1/2)T \\ 1 \; : & -\frac{DT}{4} + (k+1/2)T \leq t \leq \frac{DT}{4} + (k+1/2)T. \end{cases} \tag{46}$$

where $k \in \mathbb{N}$, and $T = 2\pi/\Omega_\mathrm{L}$ is the Larmor period. The Stokes component $\hat{S}_\mathrm{y}^\mathrm{out}$ of the linearly polarized probe beam is detected and its Fourier component at $\cos(\Omega_\mathrm{L} t)$ is measured by a lock-in amplifier. This effectively probes the atomic variable

$$\hat{X}(kT) = \frac{1}{TD}\int_{kT}^{(k+1)T} dt \frac{\hat{J}_\mathrm{z}(t)}{\sqrt{J_\mathrm{x}}} u(t)\cos(\Omega_\mathrm{L} t), \tag{47}$$

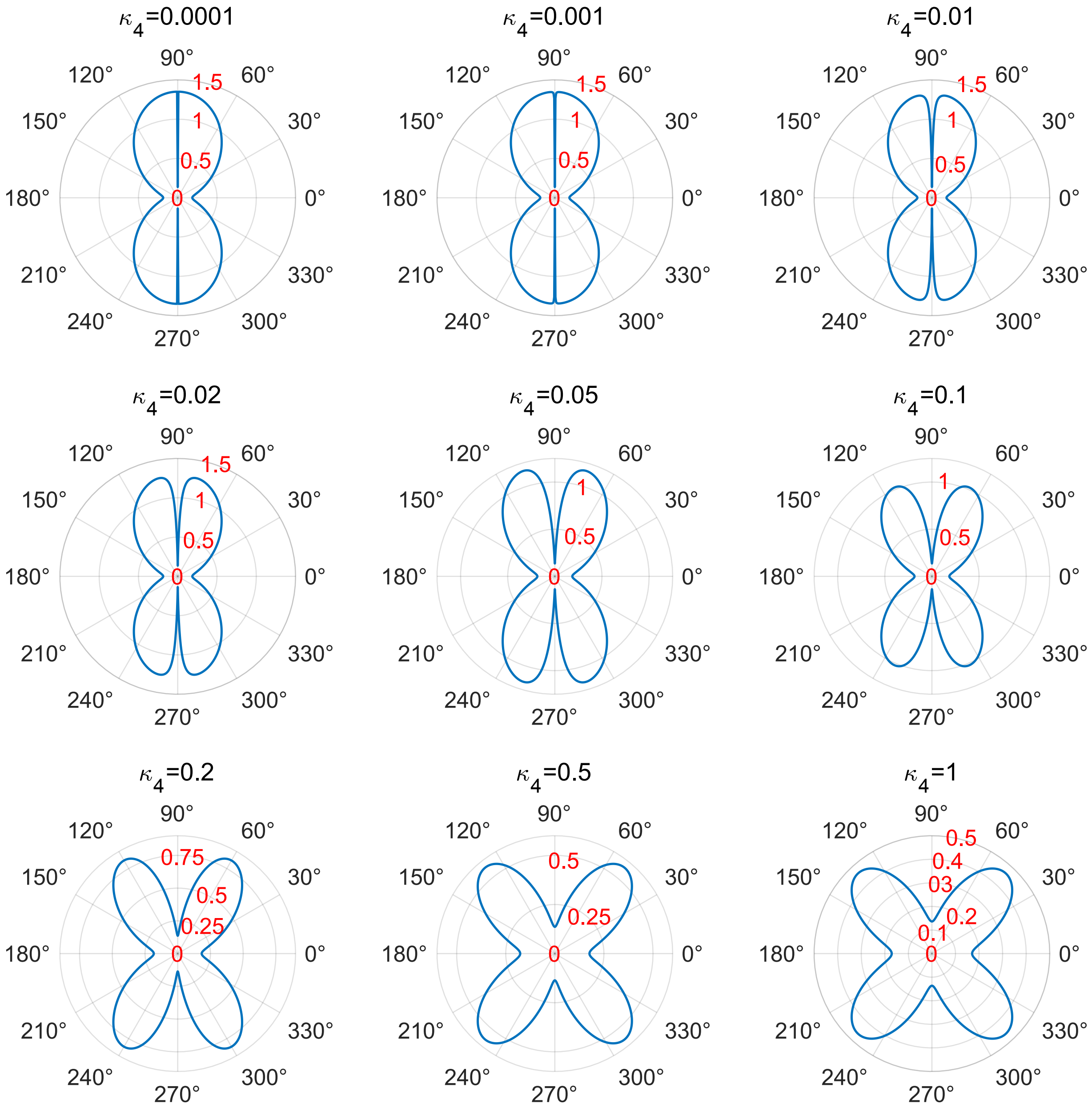

Supplementary Figure 3: Polar distribution of $\sigma^2_{\rho \mathrm{E}}$ for different coupling strengths κ_4 and fixed $\kappa_1^2 = 1.7$ and $\kappa_3^2 = 3.3$. 0.5 represents the standard quantum limit (SQL).

After a pulse train of duration $\tau = NT$ with mean photon flux $\bar{\Phi}$, the integrated optical signal is characterized by a Stokes vector with the measurement variance [1, 7, 8]

$$\mathrm{Var}(\hat{S}^{\mathrm{out}}_{\mathrm{y},\tau}) \approx \frac{\bar{\Phi}\tau}{8}\left[1 + \mathrm{Sinc}(\pi D)\right]\Big[1 + \tilde{\kappa}^2 + \frac{\tilde{\kappa}^4}{3}\frac{1 - \mathrm{Sinc}(\pi D)}{1 + \mathrm{Sinc}(\pi D)}\Big]. \tag{48}$$

Here we have introduced the effective coupling constant, $\tilde{\kappa}^2 = \frac{1}{4}\alpha^2 J_{\mathrm{x}} \bar{\Phi}\tau\left[1 + \mathrm{Sinc}(\pi D)\right]$, which becomes equal to κ^2 in the limit of vanishing duty factor D.

Disregarding the spin rotation during each pulse, we can evaluate the integrated effect of the stroboscopically-

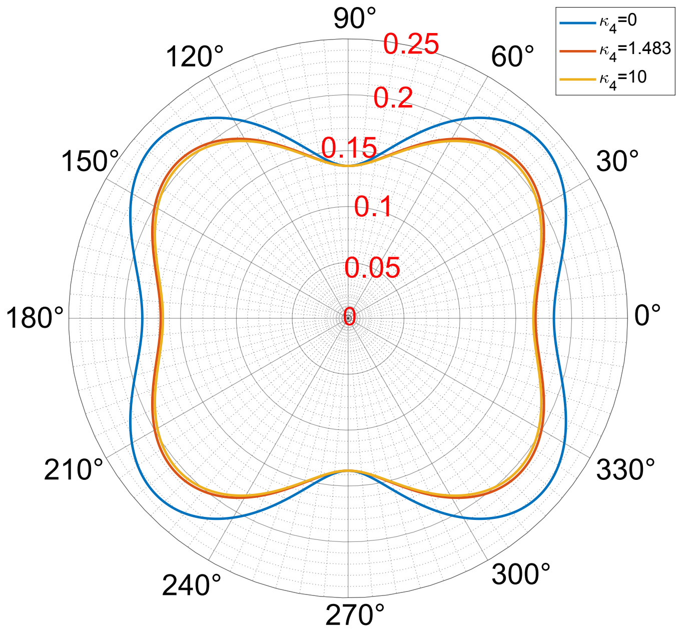

Supplementary Figure 4: Polar distribution of $(\mathrm{Var}(m_2|m_1,m_3,m_4)-\frac{1}{2})/\kappa_2^2$ for different coupling strengths κ_4 and fixed $\kappa_1^2=1.7$, $\kappa_2^2=0.81$ and $\kappa_3^2=3.3$. 0.5 represents the standard quantum limit (SQL).

modulated probing, and the conditioned squeezing is [1]

$$\begin{aligned}\xi^2 &= \frac{\mathrm{Var}\left[\hat{X}(NT)\right]\Big|_{\hat{S}_{\mathrm{y},\tau}}}{\mathrm{Var}\left[\hat{X}_0\right]} \\ &\approx 1+\tilde{\kappa}^2\frac{1-\mathrm{Sinc}(\pi D)}{1+\mathrm{Sinc}(\pi D)}-\frac{\tilde{\kappa}^2\left[1+\frac{\tilde{\kappa}^2}{2}\frac{1-\mathrm{Sinc}(\pi D)}{1+\mathrm{Sinc}(\pi D)}\right]^2}{1+\tilde{\kappa}^2+\frac{\tilde{\kappa}^4}{3}\frac{1-\mathrm{Sinc}(\pi D)}{1+\mathrm{Sinc}(\pi D)}}.\end{aligned} \tag{49}$$

$\xi_0^2=1/(1+\tilde{\kappa}^2)$ in agreement with Supplementary Equation (12) for $D=0$.

Let us now turn to estimate the effect of the spin rotation during each short probing pulse, where we expect a reduced effective coupling strength due to the shorter average projection during angular rotation.

Assume that during each optical pulse the spin rotates between the directions $-\phi$ and ϕ, where 0 denotes the direction corresponding to the observable $\hat{p}_{\mathrm{A}}$. The duty factor thus reads $D=2\phi/\pi$. Let us divide each single pulse into $2N+1$ infinitesimal duration (and hence QND) interactions in different directions, $\phi_n=\frac{n}{N}\phi$, where $n=-N,-N+1,\cdots N-1,N$. The coupling strength for each interaction is evenly distributed as $K=\frac{\tilde{\kappa}}{2N+1}$. So the evolution operator, coupling to slightly rotated atomic observables in each subinterval, becomes

$$\begin{aligned}\hat{U}_n &= e^{-iK(\hat{p}_{\mathrm{A}}\cos\phi_n+\hat{x}_{\mathrm{A}}\sin\phi_n)\otimes\hat{p}_{\mathrm{L}}} \\ &= e^{-\frac{iK^2\sin\phi_n\cos\phi_n}{2}\hat{p}_{\mathrm{L}}^2}\cdot e^{-iK\cos\phi_n\hat{p}_{\mathrm{A}}\otimes\hat{p}_{\mathrm{L}}}\cdot e^{-iK\sin\phi_n\hat{x}_{\mathrm{A}}\otimes\hat{p}_{\mathrm{L}}} \\ &= e^{\frac{iK^2\sin\phi_n\cos\phi_n}{2}\hat{p}_{\mathrm{L}}^2}\cdot e^{-iK\sin\phi_n\hat{x}_{\mathrm{A}}\otimes\hat{p}_{\mathrm{L}}}\cdot e^{-iK\cos\phi_n\hat{p}_{\mathrm{A}}\otimes\hat{p}_{\mathrm{L}}},\end{aligned} \tag{50}$$

where we use the Campbell-Baker-Hausdorff relation $e^{\hat{A}+\hat{B}}=e^{-[\hat{A},\hat{B}]/2}e^{\hat{A}}e^{\hat{B}}=e^{[\hat{A},\hat{B}]/2}e^{\hat{B}}e^{\hat{A}}$ under the condition $[\hat{A},[\hat{A},\hat{B}]]=[\hat{B},[\hat{A},\hat{B}]]=0$. The total evolution operator of a single finite duration pulse is

$$\hat{U}=\hat{U}_N\hat{U}_{N-1}\cdots\hat{U}_{-N+1}\hat{U}_{-N}. \tag{51}$$

Since $\sin\phi_{-n}=-\sin\phi_n$, we can use the Campbell-Baker-Hausdorff relation to move the term $\hat{x}_{\mathrm{A}}\otimes\hat{p}_{\mathrm{L}}$ in $\hat{U}_{-n}$ so it

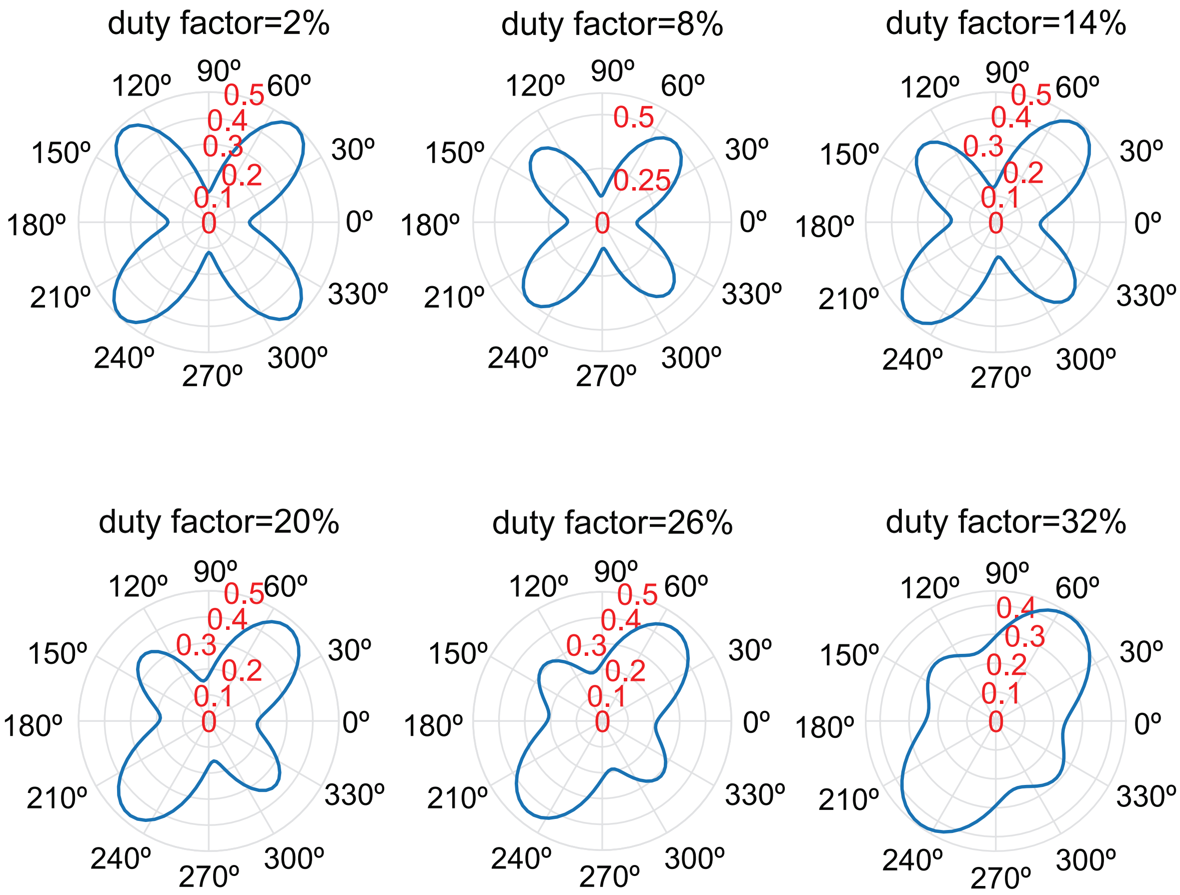

Supplementary Figure 5: Polar distribution of the $\hat{x}(\theta)$ variance for different values of the duty factor. $\kappa_1^2 = 2$, $\kappa_3^2 = 4$, $\kappa_4^2 = 2$. 0.5 represents the standard quantum limit (SQL).

cancels a similar term in $\hat{U}_n$, and we obtain

$$\hat{U} = \exp\left(-\frac{iK^2}{2}\sum_{n=-N}^{N}(\sin\phi_n\cos\phi_n)\hat{p}_{\mathrm{L}}^2\right)\cdot\exp\left(iK^2\sum_{m=1}^{N}\sin\phi_{-m}(\sum_{n=-m}^{m-1}\cos\phi_n)\hat{p}_{\mathrm{L}}^2\right)$$
$$\cdot\exp\left(-iK\sum_{n=-N}^{N}\cos\phi_n\hat{p}_{\mathrm{A}}\otimes\hat{p}_{\mathrm{L}}\right). \tag{52}$$

In the limit of $N\rightarrow+\infty$, we have

$$\hat{U} = \exp(i\kappa^2\frac{-2\phi+\sin 2\phi}{8\phi^2}\hat{p}_{\mathrm{L}}^2)\cdot\exp(-i\kappa\frac{\sin\phi}{\phi}\hat{p}_{\mathrm{A}}\otimes\hat{p}_{\mathrm{L}}). \tag{53}$$

We define $t_1 = \frac{\sin\phi}{\phi}$ and $t_2 = \frac{-2\phi+\sin 2\phi}{8\phi^2}$, and we note that when $\phi\rightarrow 0$, $t_1\rightarrow 1$ and $t_2\rightarrow 0$. $\hat{U}$ then simplifies to the $\hat{C}_z$-QND interaction described above, while a finite ϕ yields an effectively reduced coupling strength (by $\sin\phi/\phi$) and a quadratic (squeezing) effect on the optical probe, which will also alter the measurement sensitivity.

Taking into account the modification of the detection scheme due to the finite pulse duration, we obtain new

measurement operators $\hat{\Omega}_{\rm m}$

$$\begin{aligned}
\hat{\Omega}_{\rm m} &= (\hat{I}_{\rm A}\otimes\langle m|)\hat{U}(\hat{I}_{\rm A}\otimes|\Psi_{\rm L}^{in}\rangle) \\
&= \hat{I}_{\rm A}\otimes\langle m|_{\hat{x}_{\rm L}}\hat{U}\int|a\rangle\langle a|_{\hat{p}_{\rm A}}da\otimes\int\rangle_{\hat{p}_{\rm L}}dl \\
&= \hat{I}_{\rm A}\otimes\langle m|_{\hat{x}_{\rm L}}\int\int\exp(i\kappa^2t_2l^2)\cdot\exp(-i\kappa t_1al)|a\rangle\langle a|_{\hat{p}_{\rm A}}\otimes|l\rangle_{\hat{p}_{\rm L}}dadl \\
&= \hat{I}_{\rm A}\otimes\langle m|_{\hat{x}_{\rm L}}\int\int\exp(i\kappa^2t_2l^2)\cdot\exp(-i\kappa t_1al)|a\rangle\langle a|_{\hat{p}_{\rm A}}\otimes(\frac{1}{\sqrt{2\pi}}\int\exp(iml)|m\rangle_{\hat{x}_{\rm L}}dm)dadl \\
&= \frac{1}{\sqrt{2\pi}}\int\left(\int\exp(i\kappa^2t_2l^2)\cdot\exp(-i\kappa t_1al)\cdot\exp(iml)dl\right)|a\rangle\langle a|_{\hat{p}_{\rm A}}da \\
&\propto\int\exp\left(\frac{(t_1\kappa a-m)^2}{4it_2\kappa^2-2}\right)|a\rangle\langle a|_{\hat{p}_{\rm A}}{\rm d}a.
\end{aligned} \tag{54}$$

By defining $G(m,\kappa,a)=\exp[\frac{(t_1\kappa a-m)^2}{4it_2\kappa^2-2}]$, the measurement operator in the basis of $\hat{x}_{\rm A}(\theta)=\hat{p}_{\rm A}\cos\theta+\hat{x}_{\rm A}\sin\theta$'s eigenstates $|a,\theta\rangle$ with outcome m can be written as

$$\hat{\Omega}_{\rm m}\propto\int G(m,\kappa,a)|a,\theta\rangle\langle a,\theta|{\rm d}a. \tag{55}$$

This generalizes the above expression, and will now be applied to the four successive pulses, where the first measures $\hat{p}_{\rm A}$, the second second measures $\hat{x}_{\rm A}(\theta)$ to verify the variance of the past quantum state, the third measures $\hat{x}_{\rm A}$ and the fourth pulse measures $\hat{p}_{\rm A}$.

After the QND measurement pulse on $\hat{p}_{\rm A}$, the density matrix is updated as

$$\begin{aligned}
\rho &= \frac{\Omega_{m_1}\rho_0\Omega_{m_1}^\dagger}{{\rm Tr}(\Omega_{m_1}^\dagger\rho_0\Omega_{m_1})} \\
&\propto\int G(m_1,\kappa_1,a_1')\psi_{\hat{p}_{\rm A}}(a_1')G^*(m_1,\kappa_1,a_1)\psi^*_{\hat{p}_{\rm A}}(a_1)|a_1',0\rangle\langle a_1,0|{\rm d}a_1{\rm d}a_1'.
\end{aligned} \tag{56}$$

And the associated Wigner function distribution W_ρ is written as

$$\begin{aligned}
W_\rho(q,p) &= \frac{1}{\pi}\int\langle p+r,0|\rho|p-r,0\rangle e^{-2iqr}dr \\
&\propto\int G(m_1,\kappa_1,p+r)G^*(m_1,\kappa_1,p-r)\psi^*_{\hat{p}_{\rm A}}(p+r)\psi_{\hat{p}_{\rm A}}(p-r)e^{-2iqr}dr.
\end{aligned} \tag{57}$$

The distribution in the θ direction can be derived from W_ρ according to Supplementary Equation (36), with the conditional variance

$$\sigma_\rho^2=\frac{1}{2}\frac{\cos^2\theta\kappa_1^4t_1^4+4t_2\cos\theta\sin\theta\kappa_1^4t_1^2-t_1^4\kappa_1^4-2t_1^2\sin^2\theta\kappa_1^2-4t_2^2\kappa_1^4-1}{-4t_2^2\kappa_1^4-t_1^2\kappa_1^2-1}. \tag{58}$$

This variance is shown in the polar plot Fig. 2A in the main text, clearly revealing the (anti)squeezing of the $(\hat{x}_{\rm A})\hat{p}_{\rm A}$-quadrature.

The effect matrix including the third and the fourth measurement is

$$\begin{aligned}
E =&\Omega_{m_3}^\dagger\Omega_{m_4}^\dagger\Omega_{m_4}\Omega_{m_3} \\
&\propto\left[\int G^*(m_3,\kappa_3,a_3')|a_3',\frac{\pi}{2}\rangle\langle a_3',\frac{\pi}{2}|{\rm d}a_3'\right]\cdot\left[\int|G(m_4,\kappa_4,a_4)|^2|a_4,0\rangle\langle a_4,0|{\rm d}a_4\right] \\
&\quad\cdot\left[\int G(m_3,\kappa_3,a_3)|a_3,\frac{\pi}{2}\rangle\langle a_3,\frac{\pi}{2}|{\rm d}a_3\right] \\
&\propto\int G^*(m_3,\kappa_3,a_3')\left[\int G(m_3,\kappa_3,a_3)\left(\int|G(m_4,\kappa_4,a_4)|^2e^{-ia_4(a_3-a_3')}{\rm d}a_4\right)|a_3',\frac{\pi}{2}\rangle\langle a_3,\frac{\pi}{2}|{\rm d}a_3\right]{\rm d}a_3'.
\end{aligned} \tag{59}$$

Similarly, the associated Wigner function of this effect matrix is

$$W_{\mathrm{E}}(q,p) = \frac{1}{\pi}\int \langle q+r, \frac{\pi}{2}|E|q-r, \frac{\pi}{2}\rangle e^{2ipr} dr$$
$$\propto \int G^*(m_3,\kappa_3,q+r)G(m_3,\kappa_3,q-r)\left(\int |G(m_4,\kappa_4,a_4)|^2 e^{2ia_4 r} da_4\right) e^{2ipr} dr. \tag{60}$$

According to Supplementary Equation (37), the conditional variance from the effect matrix is

$$\sigma_{\mathrm{E}}^2 = \frac{1}{2}\frac{\cos^2\theta t_1^4\kappa_3^4\kappa_4^2 - 4t_1^2 t_2\cos\theta\sin\theta\kappa_3^4\kappa_4^2 + 4t_2^2\sin^2\theta\kappa_3^4\kappa_4^2 + 4t_2^2\cos^2\theta\kappa_3^2\kappa_4^4 + \cos^2\theta\kappa_3^2 + \sin^2\theta\kappa_4^2}{t_1^2\kappa_3^2\kappa_4^2}. \tag{61}$$

The past quantum state probability distribution of the atomic quadrature $\hat{x}_\theta$, conditioned on both the first, third and fourth pulse is given by

$$P(a|m_1,m_2,m_3) \propto \langle a,\theta|\rho|a,\theta\rangle\langle a,\theta|E|a,\theta\rangle \tag{62}$$

with the variance

$$\sigma_{\rho\mathrm{E}}^2 = \frac{1}{1/\sigma_\rho^2 + 1/\sigma_{\mathrm{E}}^2}. \tag{63}$$

The corresponding polar distribution is shown in Supplementary Figure 5 for different values of the duty factor.

Supplementary Note 7. Wineland criterion

To quantify how much the variance is below SQL, we use the squeezing parameter ξ_{R}^2 by the Wineland criterion [9, 10], which is essentially the ratio of the minimal angular resolving power of the spin squeezed state to that of the CSS, and has taken into account the shortening of the spin-vector due to dissipation during squeezing:

$$\xi_{\mathrm{R}}^2 = \frac{(\Delta\phi)^2}{(\Delta\phi)_{\mathrm{CSS}}^2} = \frac{\Delta(\hat{J}'_{\mathrm{y,z}})^2}{\Delta(\hat{J}_{\mathrm{y,z}})_{\mathrm{PN}}^2}\cdot\frac{|\langle J_{\mathrm{x}}\rangle|^2}{|\langle J_{\mathrm{x}}(\tau_{\mathrm{tot}})\rangle|^2}, \tag{64}$$

where $\Delta\phi$ is the minimal angular resolution for the spin squeezed state and $(\Delta\phi)_{\mathrm{CSS}}$ is the angular resolution for CSS. $\Delta\phi = \Delta\hat{J}'_{\mathrm{y,z}}/|\langle J_{\mathrm{x}}(\tau_{\mathrm{tot}})\rangle|$, $(\Delta\phi)_{\mathrm{CSS}} = \Delta(\hat{J}_{\mathrm{y,z}})_{\mathrm{PN}}/|\langle J_{\mathrm{x}}\rangle|$, where $|\langle J_{\mathrm{x}}\rangle|$ is the average spin for a perfect CSS along the x-direction, and $|\langle J_{\mathrm{x}}(\tau_{\mathrm{tot}})\rangle|$ is the average spin of the prepared CSS after a total probing time of $\tau_{\mathrm{tot}} = \tau_1 + \Delta\tau + \tau_2$ as shown in Fig.1 of main text, with τ_1 the prediction pulse duration, τ_2 the verification pulse duration, and $\Delta\tau$ the gap time in between. The ratio $|\langle J_{\mathrm{x}}\rangle|/|\langle J_{\mathrm{x}}(\tau_{\mathrm{tot}})\rangle|$ is obtained from the measurements of the population decay time T_1 with the probe on. $\Delta(\hat{J}_{\mathrm{y}})_{\mathrm{PN}}^2 = \Delta(\hat{J}_{\mathrm{z}})_{\mathrm{PN}}^2$ is the projection noise for the perfect CSS deduced from the measured spin noise of thermal state. $\Delta(\hat{J}'_{\mathrm{y}})^2$ and $\Delta(\hat{J}'_{\mathrm{z}})^2$ are the measured retrodicted variances of $\hat{J}_{\mathrm{y}}$ and $\hat{J}_{\mathrm{z}}$, respectively.

Supplementary References

[1] Heng Shen, Spin squeezing and entanglement with room temperature atoms for quantum sensing and communication. PhD thesis, University of Copenhagen (2015).

[2] D. V. Vasilyev, K. Hammerer, N. Korolev, and A. S. Sørensen, Quantum noise for Faraday light-matter interfaces. *J. Phys. B: At. Mol. Opt. Phys.* **45**, 124007 (2012).

[3] N. C. Menicucci *et al.*, Universal Quantum Computation with Continuous-Variable Cluster States. *Phys. Rev. Lett.* **97**, 110501 (2006).

[4] The conventional squeezing ellipse shows the contour of the Gaussian phase space Wigner function, and the polar plot of phase angle dependent variances is not elliptic shaped for that case. The significance of the PQS results is thus not the deviation from elliptic shapes in Supplementary Figure 1, but the fact that the distance in some (or all) mutually orthogonal directions are smaller than unity.

[5] L. B. Madsen and K. Mølmer, Spin squeezing and precision probing with light and samples of atoms in the Gaussian description. *Phys. Rev. A* **70**, 052324 (2004).

[6] K. Hammerer, K. Mølmer, E. S. Polzik, and J. I. Cirac, Light-matter quantum interface. *Phys. Rev. A* **70**, 044304 (2004).

[7] G. Vasilakis *et al.*, Generation of a squeezed state of an oscillator by stroboscopic back-action-evading measurement. *Nat. Phys.* **11**, 389-392 (2015).

[8] K. Hammerer, A. Sørensen, and E. S. Polzik, Quantum interface between light and atomic ensembles. *Rev. Mod. Phys.* **82**, 1041-1093 (2010).

[9] Wineland, D. J., Bollinger, J. J., Itano, W. M. & Heinzen, D. J. Squeezed atomic states and projection noise in spectroscopy. *Phys. Rev. A* **46**, R6797 (1992).

[10] Pezzé, L., Smerzi, A., Oberthaler, M. K., Schmied, R. & Treutlein, P. Quantum metrology with nonclassical states of atomic ensembles. *Rev. Mod. Phys.* **90**, 035005 (2018).
